# Anti-Neuronal IgG4 Autoimmune Diseases and IgG4-Related Diseases May Not Be Part of the Same Spectrum: A Comparative Study

**DOI:** 10.3389/fimmu.2021.785247

**Published:** 2022-01-14

**Authors:** Verena Endmayr, Cansu Tunc, Lara Ergin, Anna De Rosa, Rosa Weng, Lukas Wagner, Thin-Yau Yu, Andreas Fichtenbaum, Thomas Perkmann, Helmuth Haslacher, Nicolas Kozakowski, Carmen Schwaiger, Gerda Ricken, Simon Hametner, Sigrid Klotz, Lívia Almeida Dutra, Christian Lechner, Désirée de Simoni, Kai-Nicolas Poppert, Georg Johannes Müller, Susanne Pirker, Walter Pirker, Aleksandra Angelovski, Matus Valach, Michelangelo Maestri, Melania Guida, Roberta Ricciardi, Florian Frommlet, Daniela Sieghart, Miklos Pinter, Karl Kircher, Gottfried Artacker, Romana Höftberger, Inga Koneczny

**Affiliations:** ^1^ Division of Neuropathology and Neurochemistry, Department of Neurology, Medical University of Vienna, Vienna, Austria; ^2^ Department of Clinical and Experimental Medicine, Neurology Unit, University of Pisa, Pisa, Italy; ^3^ Department of Neurology, Medical University of Vienna, Vienna, Austria; ^4^ Department of Laboratory Medicine, Medical University of Vienna, Vienna, Austria; ^5^ Department of Pathology, Medical University of Vienna, Vienna, Austria; ^6^ Department of Neurology and Neurosurgery, Hospital Israelita Albert Einstein, São Paulo, Brazil; ^7^ Pediatric Neurology, Department of Pediatric and Adolescent Medicine, Medical University of Innsbruck, Innsbruck, Austria; ^8^ Department of Neurology, University Hospital St. Poelten, St. Poelten, Austria; ^9^ Department of Neurology, Christian Doppler University Hospital, Paracelsus Medical University, Salzburg, Austria; ^10^ Department of Neurology and Karl Landsteiner Institute for Neuroimmunological and Neurodegenerative Disorders, Klinik Donaustadt, Vienna, Austria; ^11^ Department of Neurology, Klinik Hietzing, Vienna, Austria; ^12^ Department of Neurology, Klinik Ottakring, Vienna, Austria; ^13^ Department of Pathology, Klinik Landstrasse, Vienna, Austria; ^14^ Center for Medical Statistics, Informatics and Intelligent Systems, Section for Medical Statistics, Medical University of Vienna, Vienna, Austria; ^15^ Division of Rheumatology, Department of Internal Medicine III, Medical University of Vienna, Vienna, Austria; ^16^ Wiener Privatklinik – Health Center, Vienna, Austria; ^17^ Department of Ophthalmology, Medical University of Vienna, Vienna, Austria; ^18^ Department of Pediatrics and Adolescent Medicine, Klinik Donaustadt, Vienna, Austria

**Keywords:** IgG4-related diseases, IgG4 autoimmune diseases, MuSK myasthenia gravis, CIDP, LGI1, Caspr2

## Abstract

**Background:**

IgG4 is associated with two emerging groups of rare diseases: 1) IgG4 autoimmune diseases (IgG4-AID) and 2) IgG4-related diseases (IgG4-RLD). Anti-neuronal IgG4-AID include MuSK myasthenia gravis, LGI1- and Caspr2-encephalitis and autoimmune nodo-/paranodopathies (CNTN1/Caspr1 or NF155 antibodies). IgG4-RLD is a multiorgan disease hallmarked by tissue-destructive fibrotic lesions with lymphocyte and IgG4 plasma cell infiltrates and increased serum IgG4 concentrations. It is unclear whether IgG4-AID and IgG4-RLD share relevant clinical and immunopathological features.

**Methods:**

We collected and analyzed clinical, serological, and histopathological data in 50 patients with anti-neuronal IgG4-AID and 19 patients with IgG4-RLD.

**Results:**

A significantly higher proportion of IgG4-RLD patients had serum IgG4 elevation when compared to IgG4-AID patients (52.63% vs. 16%, *p* = .004). Moreover, those IgG4-AID patients with elevated IgG4 did not meet the diagnostic criteria of IgG4-RLD, and their autoantibody titers did not correlate with their serum IgG4 concentrations. In addition, patients with IgG4-RLD were negative for anti-neuronal/neuromuscular autoantibodies and among these patients, men showed a significantly higher propensity for IgG4 elevation, when compared to women (*p* = .005). Last, a kidney biopsy from a patient with autoimmune paranodopathy due to CNTN1/Caspr1-complex IgG4 autoantibodies and concomitant nephrotic syndrome did not show fibrosis or IgG4^+^ plasma cells, which are diagnostic hallmarks of IgG4-RLD.

**Conclusion:**

Our observations suggest that anti-neuronal IgG4-AID and IgG4-RLD are most likely distinct disease entities.

## Introduction

In the last decade, two new groups of rare diseases emerged that are associated with the IgG4 subclass: 1) IgG4 autoimmune diseases (IgG4-AID), first appreciated as a distinct subgroup of autoimmune diseases in 2015 (1) and 2) IgG4-related diseases (IgG4-RLD), first described systematically in 2012 (2). The largest subgroup of IgG4-AID consists of anti-neuronal IgG4-AID (3), that include muscle-specific kinase (MuSK) myasthenia gravis (MG), leucine-rich glioma inactivated protein- 1 (LGI1)- and contactin-associated protein-like 2 (Caspr2)- encephalitis and autoimmune nodo-/paranodopathies with autoantibodies against contactin 1 (CNTN1)/contactin-associated protein-like 1 (Caspr1) or neurofascin-155 (NF155) (3). The diagnosis of IgG4-AID in patients presenting with disease-specific clinical symptoms (e.g., fatigable skeletal muscle weakness in MuSK-MG) is based on the detection of antigen-specific autoantibodies. IgG4-RLD is a multiorgan disease, and diagnostic criteria include organ enlargement, the presence of tissue-destructive fibrotic lesions with a storiform pattern, obliterative phlebitis, dense lymphocyte and IgG4^+^ plasma cell infiltrates and increased serum IgG4 concentrations (2, 4).

Patients with IgG4-RLD and concomitant IgG4-AID were reported in two single case reports (5, 6). They may co-occur by chance, as each of these disease groups is very rare. Epidemiological data is not widely available for these diseases, and prevalences may differ according to geographical location and disease type, but IgG4-AID are thought to have a prevalence below 5/10,000, specifically IgG4-AID with autoantibodies to MuSK (0.02/10,000), CNTN1, Caspr1 and NF155 (<0.014/10,000) (3), and LGI1 (0.0083/10,000) (7). IgG4-RLD are also rare, with estimated overall prevalence of 0.028 to 0.108/10,000 in Japan (8, 9). The question arose whether these diseases may be related (6), and we addressed this question by comparing serological, clinical, and histopathological findings in 50 patients with anti-neuronal IgG4-AID and 19 patients with IgG4-RLD to find out whether there are indications for an overlap between these diseases.

## Materials and Methods

### Patients

Sera of 50 patients (17 female, 33 male) with a clinical diagnosis of anti-neuronal IgG4 autoimmune disease (autoimmune encephalitis associated with LGI1 (n=15) or Caspr2 (n=9) autoantibodies, chronic inflammatory demyelinating polyneuropathy (CIDP) associated with anti-NF155 (n=2) or pan anti-NF155/140/186 (pan-NF, n=1), anti-CNTN1/Caspr1-complex (n=2), anti-CNTN1 (n=5) or anti-Caspr1 (n=1) autoantibodies and MuSK myasthenia gravis (n=15)) taken at the time of diagnosis or at the earliest time point available where a clear autoantibody titer was present, and sera of 53 patients with suspected neurometabolic diseases (in which IgG concentrations are considered to be unaffected by disease, 30 female, 23 male) and from 13 healthy controls (8 female, 5 male) were selected. The cohort of “suspected neurometabolic diseases” includes samples from patients with non-inflammatory diseases that were sent for diagnostic testing of metabolic diseases including Tay-Sachs disease, Sandhoff´s disease, X-linked adrenoleukodystrophy, Gaucher disease, and Fabry disease. 49/53 patients had no biochemical evidence for any type of these diseases, while 4 patients had the diagnosis of Gaucher disease. Sera of the 50 IgG4-AID patients, 53 suspected neurometabolic disease patients, and 13 healthy control patients were derived from archival blood samples that were sent for diagnostic purposes and stored at the biobank of the Division of Neuropathology and Neurochemistry, Department of Neurology, Medical University of Vienna, Austria (EK1123-2015). Archival nephelometry serum samples from the biobank of the Division of Rheumatology, Department of Internal Medicine III, Medical University of Vienna, Austria from 19 patients with IgG4-related diseases (10 female, 9 male) were analyzed retrospectively (EK559/2005). The samples were processed and stored according to standard operating procedures at the Medical University of Vienna biobank in an ISO 9001-certified environment (10).

The study was approved by the Institutional Review Boards of the Medical University of Vienna, Austria (EK 1442/2017).

### Cell-Based Assay (CBA)

Human embryonic kidney (HEK293T) cells were cultured in Dulbecco´s modified Eagle´s medium (DMEM) – high glucose supplemented with 10% fetal calf serum (FCS; Gibco), 200 mM L-glutamine (Gibco), 1x penicillin-streptomycin (Sigma) and 1x non-essential amino acids (Sigma). For transfection, cells were seeded onto poly-D lysine (PDL; Sigma) coated coverslips in tissue culture plates (p60) at a density of 1 x 10^6^ cells. After 24 hours, cells were transfected at 70 to 80% cell confluence with plasmids encoding relevant neuronal/neuromuscular antigens [LGI1-ADAM23 – courtesy of Prof. Francesc Graus, Barcelona, Spain; Caspr1 – pCMV6-Entry OriGene RC218019; CNTN1 – pCMV GeneCopoeia EXA1153-MO29; Caspr2 – courtesy of Prof. Francesc Graus, Barcelona, Spain; NF155 – pCMV6-Entry OriGene RC228652; MuSK - pIRES2-AcGFP1-MuSK (11)] using lipofectamine 2000 reagent (Invitrogen). Following 24 hours after transfection, an indirect immunofluorescent CBA was applied. Two different types of in-house CBAs were performed, depending on the antigen. For LGI1, CNTN1/Caspr1, CNTN1 and MuSK a live cell staining was performed, whereas for Caspr2 (12) and NF155 (13) the cells where fixed before they were incubated with patients’ sera. In the following both methods are described in detail.

#### Live-Labeling Cell-Based Assay

Briefly, live HEK293T cells were incubated with patients’ sera starting at a dilution of 1:40 and followed by serial dilutions, diluted in cultured medium (CNTN1/Caspr1, CNTN1, LGI1) or cultured medium supplemented with 1% bovine serum albumin (BSA) (MuSK) for 30 (CNTN1/Caspr1, CNTN1, LGI1) or 60 minutes (min) (MuSK) at 37°C. Afterwards, cells were fixed with 4% chilled paraformaldehyde (PFA; Alfa Aesar) for 10 min, permeabilized with 0.3% Triton X-100 (Merck) for 5 min (CNTN1/Caspr1, CNTN1 and LGI1 only) and incubated with a commercial antibody (anti-CNTN1, rabbit polyclonal, 1:100, Sigma #HPA070467; anti-ADAM23, rabbit polyclonal, 1:5000, Abcam #ab28304 - both diluted in 1% BSA) for 60 min at room temperature (RT). HEK293T cells were then immunolabeled with the appropriate fluorescent-conjugated Alexa Fluor^®^ secondary antibodies against human (AF594) and rabbit IgGs (AF488) (both 1:750; diluted in 1xPBS (CNTN1/Caspr1, CNTN1, LGI1) or cultured medium supplemented with 1% BSA (MuSK) for 30 min (CNTN1/Caspr1, CNTN1, LGI1) or 45 min (MuSK) at RT in the dark). For nuclear staining, 4´,6-diamidino-2-phenylindole (DAPI) was used. Cells were mounted with aqua polymount (Polysciences) onto glass slides. After drying the slides over night at 4°C in the dark, antibody binding was analyzed using an OLYMPUS BX63 fluorescence microscope.

#### Fixed Cell-Based Assay

Cultured HEK293T cells were first fixed with 4% chilled PFA for 10 min, permeabilized with 0.3% Triton X-100 for 5 min and blocked for 1.5 hours with 1% BSA to prevent unspecific protein binding. Following the blocking step, cells were incubated with patients’ sera at a dilution of 1:40, followed by serial dilutions (diluted in 1% BSA) over night at 4°C. Cells were then immunolabeled with commercial antibodies (anti-Caspr2, rabbit polyclonal, 1:5000, Abcam #ab33994; anti-c-myc, mouse monoclonal, 1:6000, Roche #11667149001 – both diluted in 1% BSA) for 30 min at RT, followed by the corresponding Alexa Fluor^®^ secondary antibodies against human (AF594) and mouse/rabbit IgGs (AF488) (both diluted 1:750 in 1xPBS). To stain the nuclei, DAPI was used and finally, the cells were mounted with aqua polymount onto glass slides. Microscopic examination and fluorescent images were performed using an OLYMPUS BX63 fluorescence microscope.

### Neuropathology

Neuropathologic analysis was performed on formalin-fixed paraffin-embedded (FFPE) tissue sections of human brain and kidney biopsy material. The brain biopsy was available from a patient with IgG4-RLD, whereas the kidney biopsy from a patient with IgG4-AID exhibiting CNTN1/Caspr1-complex autoantibodies. Tissue sections were stained with hematoxylin and eosin (H&E), Periodic acid Schiff (PAS) and silver impregnation. According to the manufacturer´s protocol, immunohistochemical stainings for the following primary antibodies were performed on an automated platform Autostainer Link 48 using the EnVision™ FLEX+ kit (Dako/Agilent) as a secondary system: CD138 (plasma cells; mouse clone B-A38; 1:200; Cell marque), IgG (heavy chains; rabbit; 1:16,000; Dako/Agilent #A0423), IgG4 (plasma cells; mouse clone HP6025; 1:500; Bio-Rad #MCA2098G). Heat-induced epitope retrieval (HIER) was performed either with target-retrieval solution low pH (Dako/Agilent) for IgG or with target-retrieval solution high pH (Dako/Agilent) for CD138 and IgG4.

Image acquisition was performed using a NanoZoomer 2.0-HT digital slide scanner C9600 (Hamamatsu Photonics, Hamamatsu, Japan).

For conventional transmission electron microscopy (EM) small samples of kidney tissue immediately were prefixed resting in toto at room temperature under fixative for a proper preservation of ultrastructure. Tissue processing was accomplished by adhering to routine standardized methods for EM, in brief fixation, dehydration and finally embedded in epoxide resin.

### Nephelometry

Human total serum IgG and subclass IgG4 concentrations were determined using particle-enhanced immune nephelometry with the BN II System (BN II Nephelometer, Siemens). The internal reference values for IgG4 were 0.03 - 2.01 g/L, and for total IgG 7-16 g/L. The published upper threshold for IgG4 in IgG4-RLD is 1.35 g/L. Serum concentrations ≥1.35 g/L were considered as elevated.

### Statistical Analysis

Due to the heteroscedastic distribution of the data and small sample size of some groups, statistical analysis with ANOVA to compare serum IgG4 concentrations between the different disease groups was considered as inappropriate. Instead, we report the mean and 95% confidence interval for each group. Nonparametric Spearman correlation was used to analyze the relationship between total IgG and IgG4 concentrations per group and between autoantibody titers and serum IgG4 concentrations. In order to test gender- and disease-dependent proportions of IgG4 elevation in patients, Fisher’s exact tests were applied. Statistical analysis was conducted using GraphPad Prism 9 and IBM SPSS Version 27.

## Results

### Patients

**Figure 1:**
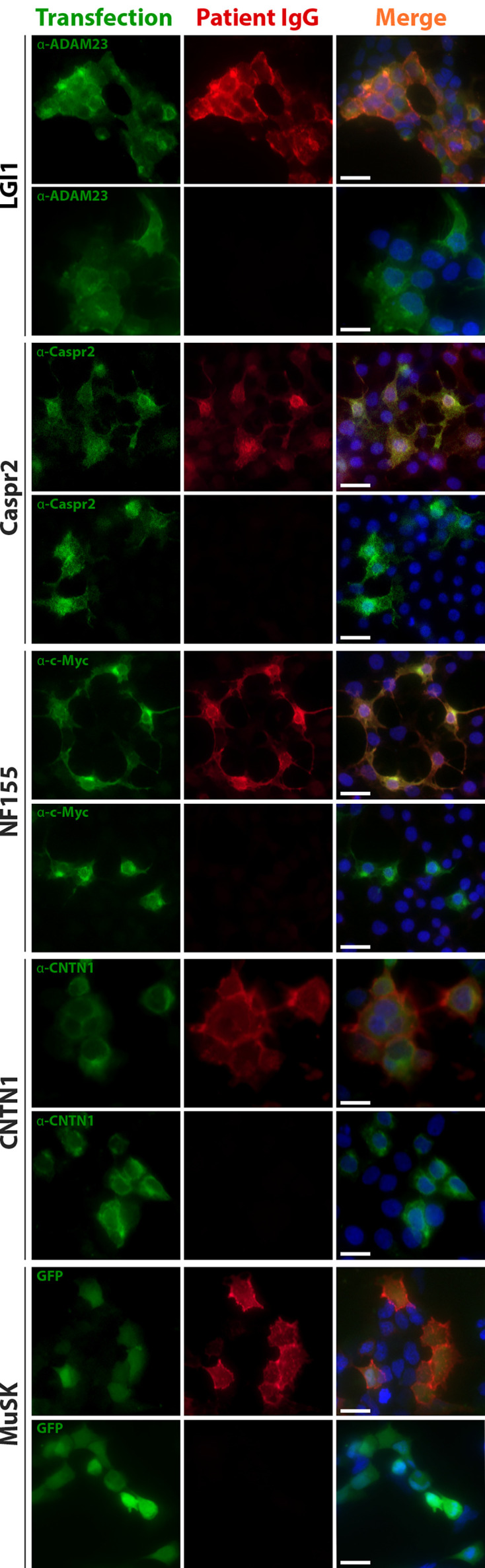
Anti-neuronal/neuromuscular autoantibodies were detected using cell-based assays. Example images of sera positive and negative for LGI1, Caspr2, NF155, CNTN1 and MuSK autoantibodies are shown. Green fluorescence indicates antigen expression by either counterstaining with commercial antibodies and secondary antibodies conjugated to AF488 or GFP co-expressed after an IRES site on the plasmid coding for MuSK. Red fluorescence indicates patient autoantibodies detected by anti-human IgG conjugated to AF594. Blue fluorescence corresponds to nuclear staining with DAPI. Scale bar , 20 µm. ADAM23 , disintegrin and metalloproteinase domain-containing protein 23; Caspr2 , contactin-associated protein-like 2; CNTN1 , contactin 1; GFP , green fluorescent protein; IgG , immunoglobulin type G; LGI1 , leucine-rich glioma inactivated protein- 1; MuSK , muscle-specific kinase; NF155 , neurofascin 155.

Anti-neuronal/neuromuscular autoantibodies were identified in all 50 patients with anti-neuronal IgG4 autoimmune disease by cell-based assays (**Figure 1**). Clinical and epidemiological data of the IgG4-AID study cohort is summarized in **Supplementary Table 1**, while **Supplementary Table 2** shows the epidemiological, clinical, serological, and histopathological data of the IgG4-RLD cohort.

### Normal Serum IgG4 Concentrations in the Majority of Patients With IgG4-AID

Elevated serum IgG4 concentrations are frequently observed in patients with IgG4-RLD, but it is unknown if it is the same for patients with anti-neuronal IgG4-AID. Total serum IgG and IgG4 concentrations were measured using nephelometry, and sera with concentrations reaching the published cut-off value of ≥1.35g/L IgG4 (14) were considered as elevated (**Table 1**). As expected, 10/19 patients with IgG4-RLD (52.63%) had elevated serum IgG4 concentrations, while the majority of healthy (84.62%) and neurometabolic controls (92.45%) had normal serum IgG4 levels (mean IgG4 concentrations 0.58 g/L ± 0.63 in the neurometabolic group versus 0.58 g/L ± 0.69 in the healthy control group). Conversely, the majority of patients with IgG4-AID (84%) had normal serum IgG4 levels. When using a Fisher´s exact test, IgG4-RLD patients were significantly more likely to display elevated IgG4, compared to patients with IgG4-AID (likelihood ratio: 8.953, *p* = .004).

**Table 1:** Number of patients with normal and elevated serum IgG4 concentrations in IgG4-AID and IgG4-RLD.

	Normal IgG4 concentrations IgG4 <1.35 g/L Patients			Elevated IgG4 concentrations IgG4 ≥1.35 g/L Patients		
Disease/Autoantibody status	Female	Male	Total	Female	Male	Total
**Healthy controls**	8 (100%)	3 (60%)	11 (84.62%)	0 (0%)	2 (40%)	2 (15.38%)
**Neurometabolic controls**	29 (96.67%)	20 (86.96%)	49 (92.45%)	1 (3.33%)	3 (13.04%)	4 (7.55%)
**IgG4-RLD**	8 (80%)	1 (11.11%)	9 (47.37%)	2 (20%)	8 (88.89%)	10 **(52.63%)**
**IgG4-AID pooled**	16 (94.12%)	26 (78.79%)	42 (84%)	1 (5.88%)	7 (21.21%)	8 **(16%)**
**LGI1 Ab pos.**	4 (100%)	8 (72.73%)	12 (80%)	0 (0%)	3 (27.27%)	3 (20%)
**Caspr2 Ab pos.**	1 (100%)	8 (100%)	9 (100%)	0 (0%)	0 (0%)	0 (0%)
**NF155/pan-NF Ab pos.**	0 (0%)	1 (33.33%)	1 (33.33%)	0 (0%)	2 (66.67%)	2 (66.67%)
**CNTN1/Caspr1, CNTN1, Caspr1 Ab pos.**	0 (0%)	6 (75%)	6 (75%)	0 (0%)	2 (25%)	2 (25%)
**MuSK Ab pos.**	11 (91.67%)	3 (100%)	14 (93.33%)	1 (8.33%)	0 (0%)	1 (6.67%)

Values indicate number of patients (female, male and total) per group (normal and elevated IgG4), percentages indicate percent of females, males or total of both groups (normal and elevated IgG4).

Ab, antibody; Caspr1, contactin-associated protein-like 1; Caspr2, contactin-associated protein-like 2; CNTN1, contactin 1; IgG4, immunoglobulin type G subclass 4; IgG4-AID, IgG4 autoimmune disease; IgG4-RLD, IgG4-related disease; LGI1, leucine-rich glioma inactivated protein- 1; MuSK, muscle-specific kinase; NF155, neurofascin 155; pan-NF Ab pos, positive for pan neurofascin antibodies; pos, positive.

Bold values indicate the percentage of elevated serum IgG4 concentration in IgG4-RLD and in IgG4-AID.

**Figure 2:**
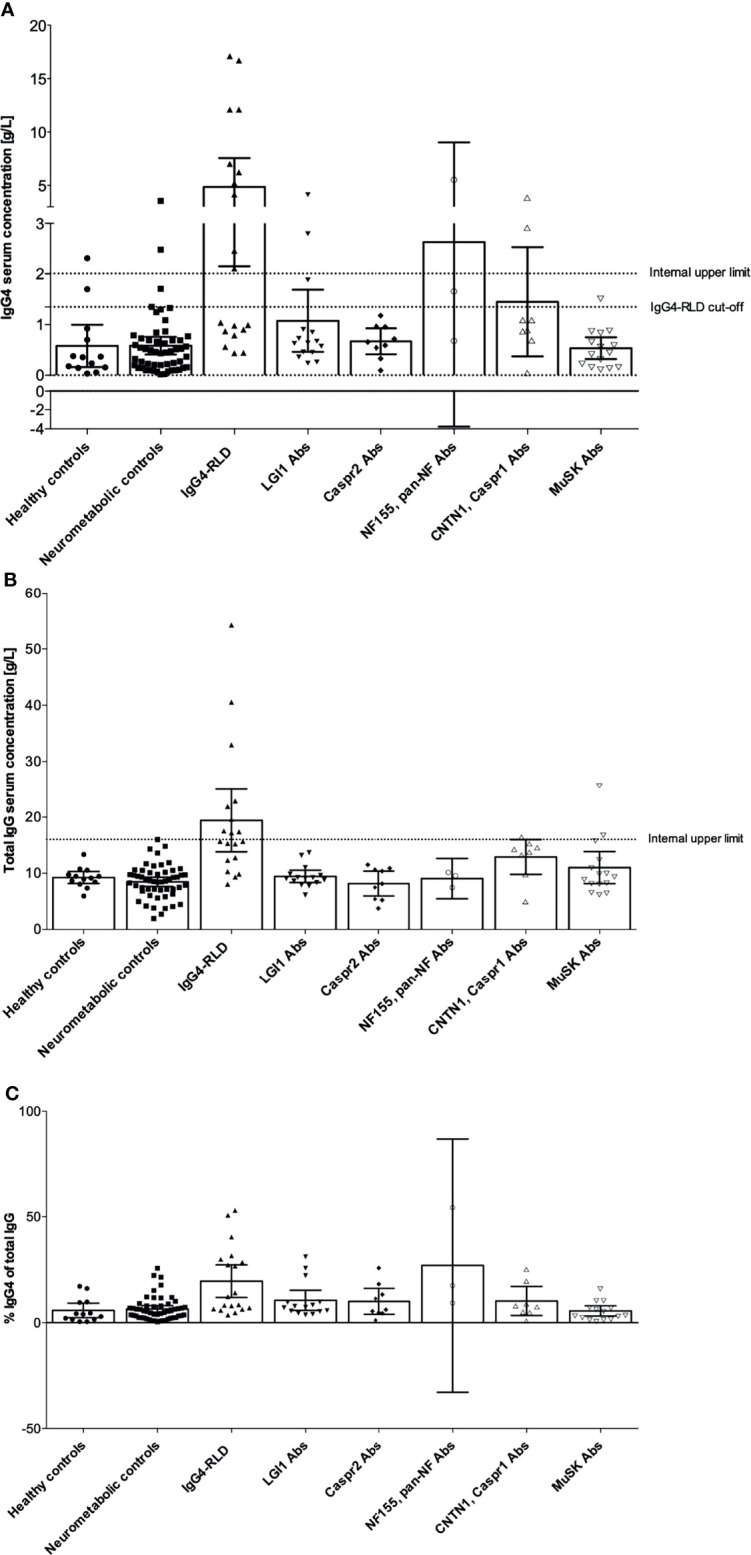
Serum IgG4 and total IgG concentrations in patients with IgG4 autoantibodies, IgG4-RLD, neurometabolic and healthy controls. IgG4 concentrations were obtained by nephelometry. **(A)** Serum IgG4 concentrations. The internal upper limit for IgG4 concentrations is indicated as a line at 2.01 g/L, the official cut-off for elevated IgG4 concentrations in IgG4-RLD is indicated as a line at 1.35 g/L. **(B)** Total serum IgG concentrations. The internal upper limit for total IgG is indicated as a line at 16 g/L. **(C)** Percent IgG4 of total IgG. Bar graphs indicate mean and error bars indicate 95% CI. Abs, antibodies; Caspr1, contactin-associated protein-like 1; Caspr2, contactin-associated protein-like 2; CNTN1, contactin 1; IgG, immunoglobulin type G; IgG4, immunoglobulin type G subclass 4; IgG4-RLD, IgG4-related disease; LGI1, leucine-rich glioma inactivated protein- 1; MuSK, muscle-specific kinase; NF155, neurofascin 155.

The highest absolute serum IgG4 concentrations (**Figure 2A**) were observed in patients with IgG4-RLD (up to 17.1 g/L). Eight patients with LGI1, CNTN1/Caspr1-complex, NF155 or pan-NF and MuSK autoantibodies had elevated serum IgG4 concentrations, but these were in a similar range as in six of the healthy and neurometabolic controls.

**Figure 3:**
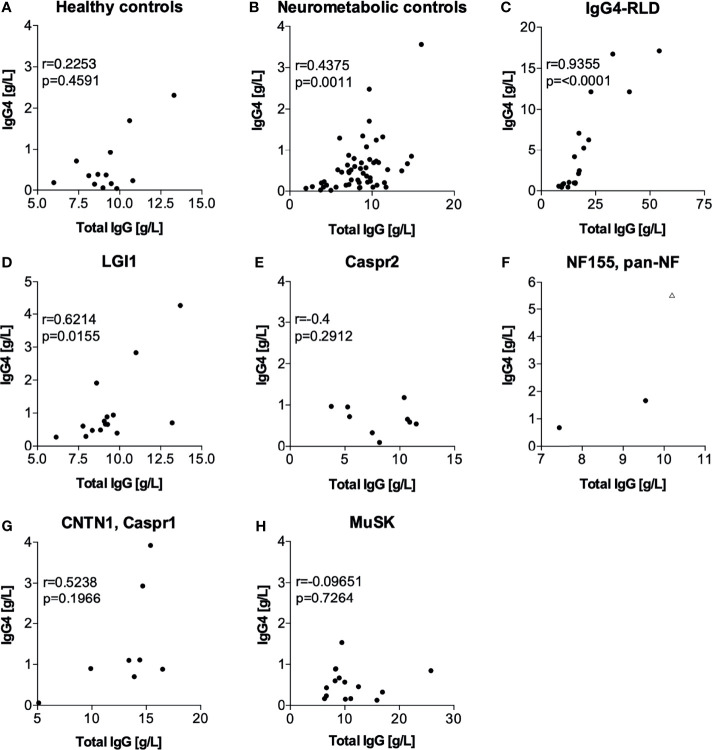
Correlation between serum IgG4 and total IgG concentrations (statistical analysis with Spearman correlation in datasets with at least 3 datapoints). **(A)** healthy controls, **(B)** neurometabolic controls, **(C)** IgG4-RLD, **(D)** LGI1, **(E)** Caspr2, **(F)** NF155, **(G)** CNTN1, Caspr1 and **(H)** MuSK. In **(F)**, the triangular data point indicates a patient that was excluded from the statistical analysis due to the presence of pan-NF antibodies instead of NF155 antibodies and severe concomitant autoimmune diseases. Caspr1, contactin-associated protein-like 1; Caspr2, contactin-associated protein-like 2; CNTN1, contactin 1; IgG, immunoglobulin type G; IgG4, immunoglobulin type G subclass 4; IgG4-RLD, IgG4-related disease; LGI1, leucine-rich glioma inactivated protein- 1; MuSK, muscle-specific kinase; NF155, neurofascin 155.

Patients with IgG4-RLD also had elevated total serum IgG concentrations (**Figure 2B**) and increased relative IgG4 concentrations (**Figure 2C**). We reasoned that in these patients, serum IgG4 contributed substantially to the total IgG concentrations, and we found a significant correlation between total IgG and IgG4 (r=0.9355, *p* <.0001, **Figure 3C**). Significant correlations were also observed in the neurometabolic controls (**Figure 3B**) and in patients with LGI1 autoantibodies (**Figure 3D**) with elevated IgG4 concentrations, but their total serum IgG concentrations were in the normal range (**Figure 2B**). No correlations were found in the other groups (**Figures 3A, E–H**).

### Serum IgG4 Concentrations Were Higher in Males Than in Females

**Figure 4:**
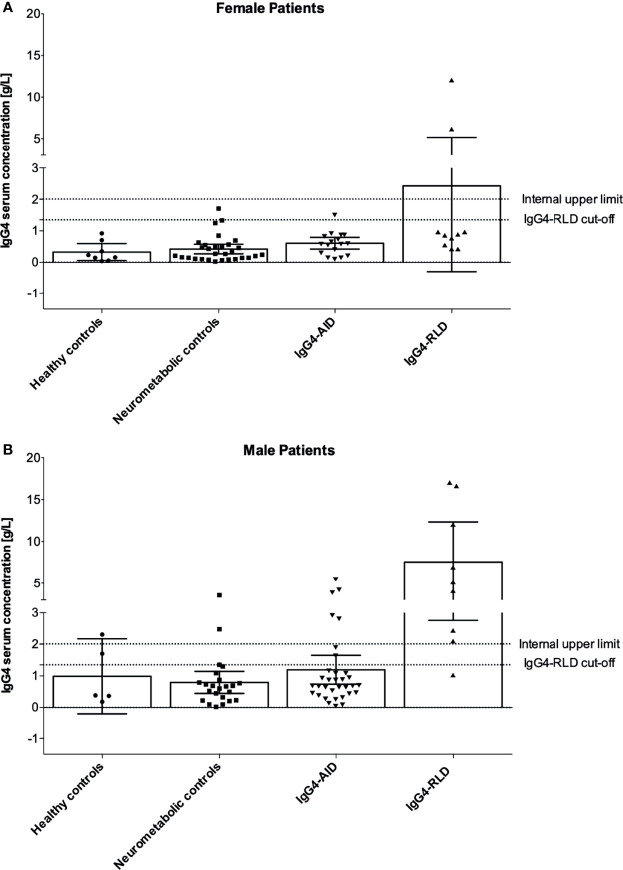
Gender-specific serum IgG4 concentrations. **(A)** Serum IgG4 concentrations in female patients, **(B)** Serum IgG4 concentrations in male patients. Bar graphs indicate mean, error bars indicate 95% CI. IgG4, immunoglobulin type G subclass 4; IgG4-AID, IgG4 autoimmune disease; IgG4-RLD, IgG4-related disease.

We observed that IgG4 was more frequently elevated in males than in females (**Table 1**). In IgG4-RLD, IgG4 was elevated in 88.89% of males but only in 20% of females. These different proportions were significant in a Fisher´s exact test (likelihood ratio: 10, *p* = .005). Elevated IgG4 was also observed rather in male patients with IgG4-AID (21.21%) than in females (5.88%) (likelihood ratio: 2.255, *p* = .237). The absolute IgG4 concentrations in all disease groups were higher in males than in females (**Figure 4**), with maximum values, respectively for male and female, of 17.1 g/L vs. 12.1 g/L (IgG4-RLD), 5.55 g/L vs. 1.53 g/L (IgG4-AID), 3.56 g/L vs. 1.71 g/L (neurometabolic controls) and 2.31 g/L vs. 0.92 g/L (healthy controls).

### Serum IgG4 Levels Did Not Correlate With Anti-Neuronal/Neuromuscular Autoantibody Titers/Scores

We further addressed whether serum IgG4 concentrations were associated with anti-neuronal/neuromuscular autoantibody titers/scores but found no overall correlation (**Supplementary Figure 1**). Interestingly, one patient with pan-NF antibodies showed a highly elevated relative and absolute serum IgG4 concentration (5.55 g/L, 54% IgG4 of total IgG) and an exceptionally high serum antibody titer of 1:40,960. Nevertheless, this patient had severe comorbidities for 11 years including multiple sclerosis and Grave’s disease, and was treated with intravenous immunoglobulin (IVIg), plasma exchange (PLEX) and Interferon β1a and was therefore excluded as outlier from the statistics.

### Lack of Overlap Between IgG4-AID and IgG4-RLD

**Figure 5:**
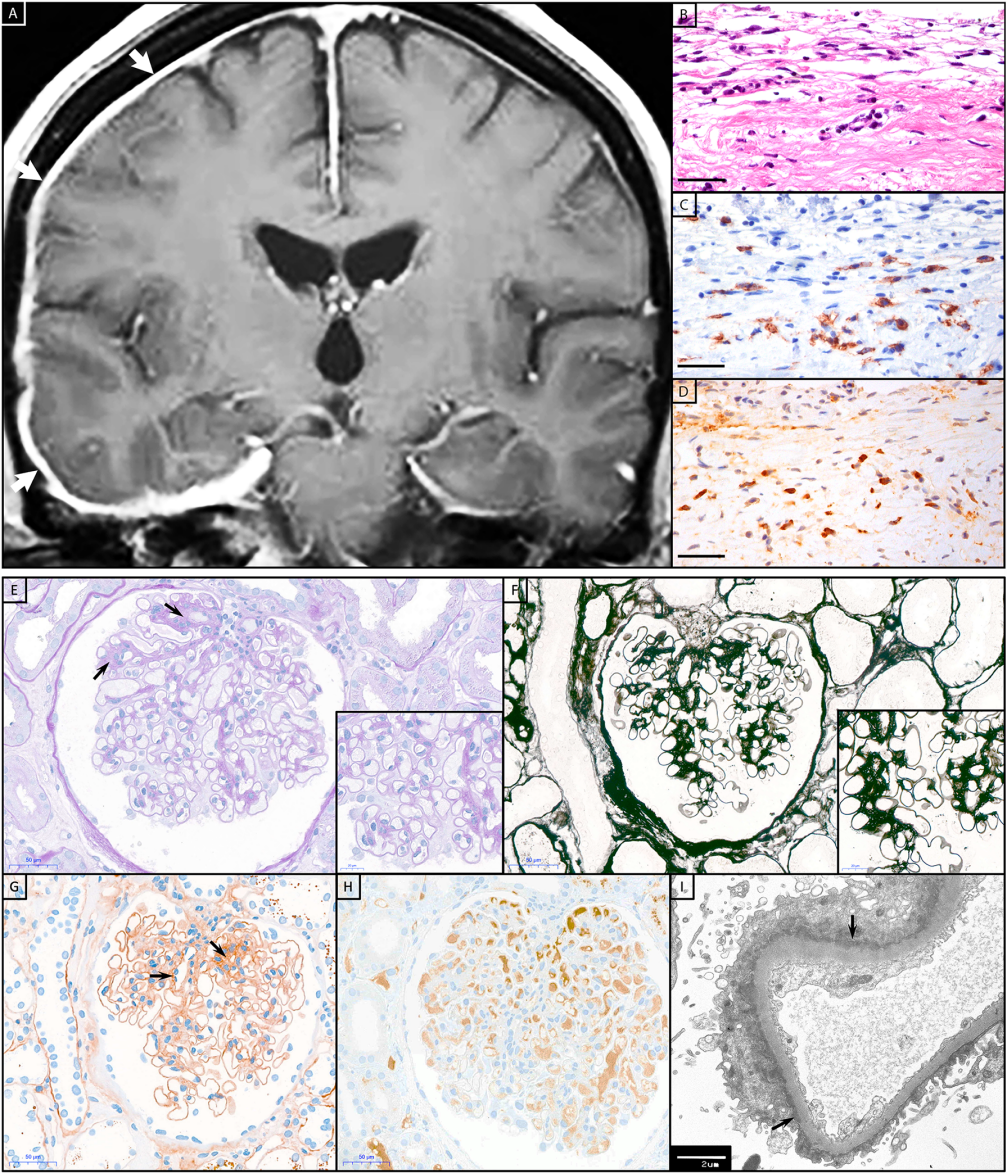
Histopathology of IgG4-RLD and IgG4-AID patients. Patient with IgG4-RLD: **(A)** MRI shows a right-sided thickening and increased contrast agent uptake of the pachymeninges (white arrows). A biopsy from the meninges reveals fragments of dura with **(B)** prominent fibrosis and infiltration with **(C)** numerous CD138^+^ and **(D)** IgG4^+^ plasma cells, compatible with IgG4-related pachymeningitis. Patient with IgG4-AID: A kidney biopsy from a patient with CNTN1/Caspr1-complex autoantibodies and acute kidney failure, nephrotic syndrome, hypoalbuminemia and microhematuria. **(E)** Glomerulus with mild segmental fibrous mesangial expansion (arrows), somewhat thickened capillary basal membranes, without hypercellularity (PAS stain). **(F)** Silver stain with smooth capillary loop basal membranes. **(G)** Immunohistochemistry for IgG with global finely granular peripheral and segmental mesangial positive deposits (arrows). **(H)** Negative immunohistochemistry for IgG4. **(I)** Electron microscopy with numerous small sub-epithelial electron-dense deposits with flattening of podocytic foot processes (arrows). Scale bar **(B–D)**, **(E–H)** = 50 µm; scale bar inset in **(E, F)** = 20 µm; scale bar **(I)** = 2 µm.

16% of the IgG4-AID patients showed increased serum IgG4 concentrations (**Table 1**). Their clinical and histopathological data were analyzed for key symptoms of IgG4-RLD that are considered as diagnostic criteria for IgG4-RLD (2, 4), specifically 1) organ enlargement, 2) tumefactive lesions, 3) fibrosis or 4) IgG4^+^ plasma cell infiltrates (**Figures 5A–D**). None of the IgG4-AID patients, including those with elevated IgG4 serum levels, fulfilled these diagnostic criteria for IgG4-RLD.

**Figure 6:**
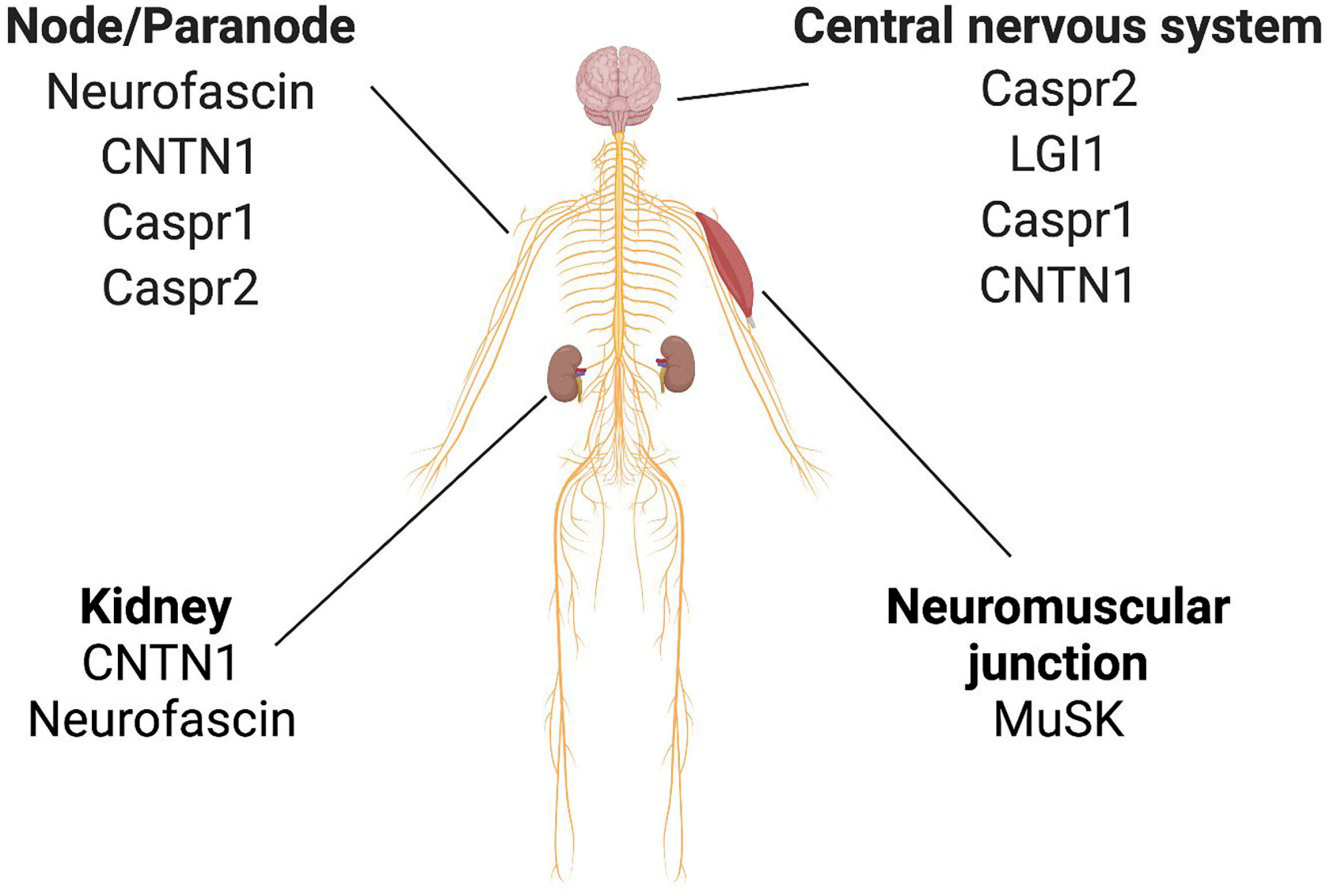
Expression of selected neuronal/neuromuscular antigens that are targeted by pathogenic IgG4 autoantibodies (in a selection of relevant organs). Caspr1, contactin-associated protein-like 1; Caspr2, contactin-associated protein-like 2; CNTN1, contactin 1; LGI1, leucine-rich glioma inactivated protein- 1; MuSK, muscle-specifific kinase.

Neuronal proteins such as CNTN1 and neurofascin186 are also expressed on podocytes in the kidney [**Figure 6**, (15, 16)]. Accordingly, patients with chronic inflammatory demyelinating polyradiculoneuropathy (CIDP) may develop membranous glomerulonephritis (17–25). Therefore, the kidney is also a target for IgG4 autoantibodies in these patients (26, 27). Histopathological characteristics of IgG4-RLD were investigated in a kidney biopsy of one CIDP patient (male, 54 years) with CNTN1/Caspr1-complex autoantibodies, nephrotic syndrome and microhematuria.

The histopathological workup of the kidney biopsy (**Figures 5E–I**) showed minimal membranous glomerulopathy class I with normal glomeruli by light microscopy but mesangial immune deposits by electron microscopy. Nevertheless, neither IgG4 deposits, fibrosis nor IgG4^+^ plasma cell infiltrates were observed in this biopsy.

We further addressed whether IgG4-RLD patients display anti-neuronal/neuromuscular autoantibodies. Sera from nine patients with IgG4-RLD were available and were tested in tissue-based assays on rat brain for the presence of anti-neuronal/neuromuscular autoantibodies. All sera were negative in the tissue-based assay (**Supplementary Figure 2**).

## Discussion

IgG4-AID and IgG4-RLD are two groups of rare diseases associated with IgG4. To date, it has not been systematically analyzed whether these two groups are part of the same disease spectrum. To the best of our knowledge, we are the first to answer this question by performing a comparative analysis between neuronal/neuromuscular cell surface IgG4 autoimmunity and IgG4-RLD to see if they share diagnostic characteristics.

As a result, we could not observe any indication for an overlap between anti-neuronal IgG4-AID and IgG4-RLD, as firstly, 84% of patients with IgG4-AID had normal IgG4 levels, and serum IgG4 concentrations did not correlate with antigen specific autoantibody titers/scores and, secondly, we could not identify clinical or histopathological indications for IgG4-RLD, while, thirdly, a substantial fraction of IgG4-RLD patients (52.63%) had elevated serum IgG4 concentrations, but all IgG4-RLD patients were negative for anti-neuronal/neuromuscular autoantibodies. In a tissue-based assay, which represents a broad screening method for autoantibodies against a variety of neuronal and glial epitopes, no reactivity was found in the sera from patients with IgG4-RLD. However, we did not test for the presence of non-neuronal IgG4 autoantibodies, which therefore cannot be ruled out.

In the healthy and neurometabolic controls as well as in the IgG4-AID patient group, few individuals showed elevated serum IgG4 concentrations. This has to be expected, since IgG4 concentrations vary and may temporarily/seasonally increase due to a change in immune status, e.g. due to infections or allergy. IgG4 levels are known to be higher in males than in females (28, 29), which we also observed, with most male IgG4-RLD patients (88.89%) presenting with elevated serum IgG4 in contrast to only 20% of female IgG4-RLD patients.

Our patients fulfilled clinical, serological and/or histopathological characteristics of IgG4-RLD (30). In general, approximately 70% of patients with IgG4-RLD present with elevated serum IgG4 (14), while in our study 52.63% of IgG4-RLD patients had elevated serum IgG4. This difference is most likely due to the relatively high proportion of female patients in our cohort (10 females, 9 males, or 1.11:1 ratio F:M), while in general males are predominantly affected with ratios of up to 1:5.7 (F:M). If only male patients are considered, 88.89% of patients in our cohort had elevated serum IgG4, which is more similar to the published 70%. This predisposition to elevated serum IgG4 may pose male patients at increased risk for suffering from IgG4-RLD, which accord to the male predominance of IgG4-RLD (**Table 2**). Elevated IgG4 levels may offer better protection from classical IgG1/IgG3 mediated autoimmune diseases such as AChR myasthenia gravis (MG), which is indeed more frequent in women (43). Interestingly, AChR-IgG4 protected from MG in an animal model (44). Nevertheless, pathogenic IgG4 autoantibodies cause pemphigus and MuSK myasthenia gravis, which have a clear female predominance (**Table 2**). However, we found that most MuSK MG patients had normal/low serum IgG4 concentrations, suggesting that the total IgG4 concentrations are unrelated to MuSK antibody pathogenicity. Our observations were in line with another study that describes normal IgG4 levels in the majority of pemphigus patients (24/27 pemphigus vulgaris and 13/16 pemphigus foliaceus patients had normal IgG4 levels) (45).

**Table 2:** Summary of IgG4-AID vs. IgG4-RLD.

Disease aspects	IgG4 autoimmune diseases (IgG4-AID)	IgG4-related diseases (IgG4-RLD)	References
Prevalence†, (per 10,000)	<0.0001 – 5	0.028 – 0.108 (Japan)	(3, 8, 9, 31)
Gender predisposition†,	MuSK MG, pemphigus, thrombotic thrombocytopenic purpura: female predominance LGI1, Caspr2 encephalitis, CIDP (NF155, CNTN1): male predominance	Male predominance	(32–36)
Affected organs	Currently known: nervous system, kidneys, blood, skin and mucosa	All organs/multiorgan, often in salivary glands, lymph nodes and pancreas	(3, 37)
Fibrosis	No	Yes^‡^	(37)
Tissue infiltrates of IgG4^+^ lymphocytes	No	Yes^‡^	(37)
Organ enlargement, tumor-like mass formation in affected organ	No	Yes, often in lacrimal glands, orbits, major salivary glands, pancreas, bile ducts, retroperitoneum, lungs, kidneys, aorta, pachymeninges and thyroid gland	(37)
Suspected HLA risk loci	HLA-DRB1*14, HLA-DQB1*05, HLA-DRB1*14-DQB1*05, HLA-DRB1*15, HLA-DRB1*04, DRB1*03 protective	HLA-DRB1*04:05, HLA-DQB1*04:01, HLA-A, HLA-C, HLA I, HLA-DQB1*03:02, HLA-B*07, HLA-B*08, HLA-DRB1*15	(38, 39)
IgG4 concentrations	Normal	Elevated (≥1.35g/L in 70% of patients)^‡^	(14)
Autoantigen-specific IgG4	In 100% of cases^‡^	In a subset of patients	(3, 40)
Location of known IgG4 autoantigen	Extracellular	Intracellular and extracellular	(3, 40)
Role of IgG4	Directly pathogenic^‡^	Unclear	(3, 40)
Pathogenic mechanism of IgG4	IgG4 blocks protein-protein interactions	Unknown	(3, 40)
Treatment response	Moderate success of corticosteroid treatment. B cell depletion beneficial especially in treatment resistant patients	Moderate success of corticosteroid treatment. B cell depletion beneficial especially in treatment resistant patients	(3, 41, 42)

^†^Few epidemiological data available, the values shall be considered as estimates. ^‡^characteristics are considered as pathognomonic. Caspr2, contactin-associated protein-like 2; CIDP, chronic inflammatory demyelinating polyneuropathy; CNTN1, contactin 1; HLA, human leucocyte antigen; IgG4, immunoglobulin type G subclass 4; LGI1, leucine-rich glioma inactivated protein- 1; MG, myasthenia gravis; MuSK, muscle-specific kinase; NF155, neurofascin 155.

Importantly, antigen-specific IgG4 directly cause neurological symptoms of IgG4-AID (39), while the pathogenic mechanisms of IgG4 in IgG4-RLD are currently not well understood. So far, only very few target antigens have been described in IgG4-RLD (40), but these are mostly located intracellularly. For example, antibodies in IgG4-related autoimmune pancreatitis (IgG4-AIP), a form of IgG4-RLD, may target annexin A11 (46), which is located in the nucleus (47). Passive transfer of patient IgG1 and IgG4 from patients with IgG4-AIP to experimental animals^1^ showed that both IgG1 and IgG4 induced pancreatic injury, but IgG4 also led to significant reduction of necrosis when co-injected with IgG1 (48). Similar observations were made with IgG4 against annexin A11, which blocked binding of IgG1 (46). Therefore, at the moment the role of IgG4 in IgG4-RLD remains elusive.

Interestingly, some overlap of IgG4-AID and IgG4-RLD is currently discussed for anti-neutrophil cytoplasmic autoantibodies (ANCA) associated vasculitis (granulomatosis with polyangiitis; GPA; also called Wegener’s granulomatosis). GPA is characterized by antigen-specific IgG4, IgG3 or IgG1 against proteinase 3 (PR3; surface antigen) or myeloperoxidase (MPO; intracellular protein) in neutrophils and monocytes, increased levels of IgG4^+^ plasma cells, fibrosis and sometimes elevated serum IgG4 levels (49–52). The patients may additionally present with IgG4-RLD (53–55), but also with PLA2R autoantibodies (56), which are found in another IgG4-AID, PLA2R-antibody positive membranous nephropathy (57). Nevertheless, it is still unclear whether GPA belongs to the IgG4-AID, as the pathogenicity of IgG4-PR3 has not yet been demonstrated by passive transfer of IgG4 to experimental animals.

To date it is not known what steers the autoimmune response in IgG4-AID towards the production of pathogenic IgG4. Few studies are available that provide evidence for the underlying etiology and immunopathology. Under physiological conditions, IgG4 and IgE production is stimulated by interleukin-4 (IL-4) and interleukin-13 (IL-13), but additional stimulation with interleukin-10 (IL-10) is considered as decisive factor for IgG4 class switch (58–61). IL-10 is an anti-inflammatory cytokine, produced by regulative T cells (Tregs) and regulative B cells/B10 cells (62–65) and IL-10 secreting B cells predominantly produced IgG4 in one study (66). Therefore, IL-10 may also play a role in the production of pathogenic IgG4 in patients, together with further factors that may predispose to develop autoimmunity. Susceptibility to develop autoimmune diseases is also associated with genetic variations in the HLA gene locus (67). Distinct HLA variants were described to induce either a pro-inflammatory or a tolerogenic immune response, the latter included also increased production of IL-10 (68). We hypothesize that IgG4-AID associated HLA variants (e.g. HLA-DQB1*05, HLA-DRB1*14) may contribute to the susceptibility to IgG4-AID (39), perhaps *via* altered IL-10 production. MuSK myasthenia gravis patients with HLA-DRB1*14 presented with increased serum levels of IgG4 autoantibodies and IL-10 concentrations (69), and IL-10 also plays a role in animal models of pemphigus vulgaris (70–72) and MuSK myasthenia gravis (73). Another open question is what could induce the production of autoantibodies in IgG4-AID. IgG4 has anti-inflammatory properties and is thought to play a role in the resolution of inflammation after prolonged exposure to antigen, e.g., in the context of allergy or helminth infection (74–76). Perhaps pathogenic IgG4 autoantibodies are the result of a tolerogenic immune response after prolonged stimulation with an environmental antigen that shows structural similarities to a self-antigen. There is evidence supporting this idea from the skin blistering disease Fogo selvagem, an endemic form of pemphigus vulgaris. Here, IgG4 subclass autoantibodies against the keratinocyte antigen desmoglein 1 are thought to arise after stimulation with an antigen present in the saliva of the sandfly (Lutzomyia longipalpis) as a result of a cross-reaction (77). Overall, the immunobiology and etiology of IgG4-AID are not well characterized yet and are also the topic of an ongoing series of reviews (3, 39, 41).

## Conclusion

IgG4-AID and IgG4-RLD are most likely distinct disease groups. Due to their low disease prevalences, comparative data to characterize these diseases are limited. In our study we provide three relevant findings, 1) a significantly higher proportion of IgG4-RLD patients (52.63%) had elevated serum IgG4 concentrations compared to IgG4-AID (16%), 2) IgG4-AID patients with elevated IgG4 did not meet the diagnostic criteria of IgG4-RLD and their autoantibody titers/scores did not correlate with serum IgG4 concentrations, while 3) patients with IgG4-RLD were negative for anti-neuronal/neuromuscular IgG4 autoantibodies. Furthermore, male IgG4-RLD patients presented significantly more frequently with elevated serum IgG4 compared to female patients.

In summary, our data do not support clinical or histopathological commonalities between IgG4-AID and IgG4-RLD, suggesting that they are in fact unrelated. Further studies on IgG4-AID and IgG4-RLD will lead to a better understanding of these diseases.

## Data Availability Statement

The original contributions presented in the study are included in the article/**Supplementary Material**. Further inquiries can be directed to the corresponding author.

## Ethics Statement

The study was approved by the Institutional Review Boards of the Medical University of Vienna, Austria (EK 1442/2017). Written informed consent to participate in this study was provided by the participants’ legal guardian/next of kin.

## Author Contributions

VE: Data Curation, Formal Analysis, Investigation, Visualization, Writing – Original Draft Preparation, Writing – Review & Editing. CT, LE, AR, RW, LW, T-YY, AF, TP, HH, CS, GR, SK, LD, CL, DdS, K-NP, GM, SP, WP, AA, MV, MM, MG, RR, DS, MP, KK, and GA: Investigation, Writing – Review & Editing. NK: Investigation, Visualization, Writing – Review & Editing. SH and FF: Data Curation, Formal Analysis, Investigation, Writing – Review & Editing. RH: Conceptualization, Funding Acquisition, Investigation, Project Administration, Resources, Supervision, Visualization, Writing – Original Draft Preparation, Writing – Review & Editing. IK: Conceptualization, Data Curation, Formal Analysis, Funding Acquisition, Investigation, Project Administration, Resources, Supervision, Visualization, Writing – Original Draft Preparation, Writing – Review & Editing. All authors have approved the final version of the manuscript.

## Funding

This work was supported by grants from the Austrian Science Fund (FWF), project number T996-B30, SYNABS project number I4685-B, DOC33-B27 and the Austrian Society of Neurology (Österreichische Gesellschaft für Neurologie). **Figure 6** was created with BioRender software (license IK).

## Conflict of Interest

LD received a grant from Fleury Laboratory for the Brazilian Autoimmune Encephalitis Project without personal compensation. CL served as a consultant for Roche. K-NP received a travel grant from Merck. RH reports speakers’ honoraria from Novartis and Biogen. The Medical University of Vienna (Austria; employer of Dr. Höftberger) receives payment for antibody assays and for antibody validation experiments organized by Euroimmun (Lübeck, Germany).

The remaining authors declare that the research was conducted in the absence of any commercial or financial relationships that could be construed as a potential conflict of interest.

## Publisher’s Note

All claims expressed in this article are solely those of the authors and do not necessarily represent those of their affiliated organizations, or those of the publisher, the editors and the reviewers. Any product that may be evaluated in this article, or claim that may be made by its manufacturer, is not guaranteed or endorsed by the publisher.

## Footnotes

1 If the pathogenic mechanism depends on interaction with other parts of the immune system, precautions such as injection of human complement along with the antibodies are necessary as human and animal immune systems are not always compatible.
