# Supplementary material for: Anti-Neuronal IgG4 Autoimmune Diseases and IgG4-Related Diseases May Not Be Part of the Same Spectrum: A Comparative Study
Source: Front Immunol. 2022 Jan 14;12:785247. doi: 10.3389/fimmu.2021.785247 (PMC8795769; doi:10.3389/fimmu.2021.785247)
Supplement: Supplementary file 1 [file DataSheet_1.pdf]

Supplementary Material

Supplementary Figure 1

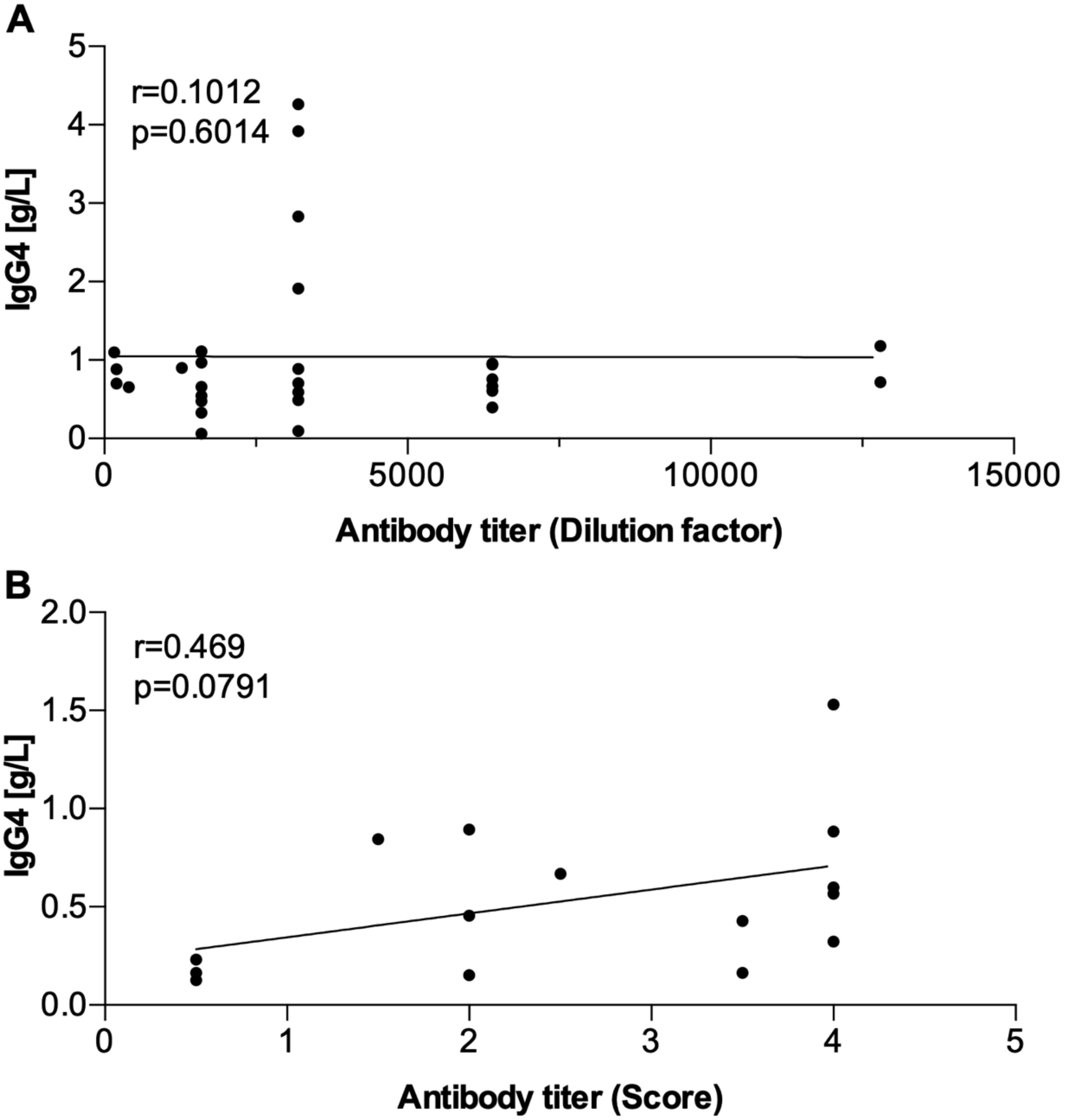

Supplementary Figure 1: No apparent correlation between serum IgG4 concentrations and IgG4 autoantibody titers/scores could be observed. (A) LGI1, Caspr2, NF155, CNTN1/Caspr1 antibody titers are expressed as dilution factor at which autoantibodies were still detectable. **(B)** MuSK antibody titers are determined by visual scoring of immunofluorescent signal in cell-based assay, from 0 = negative to 4 = highest intensity. Nonparametric Spearman correlation with two-tailed p-value. IgG4 = immunoglobulin type G subclass 4.

Supplementary Figure 2

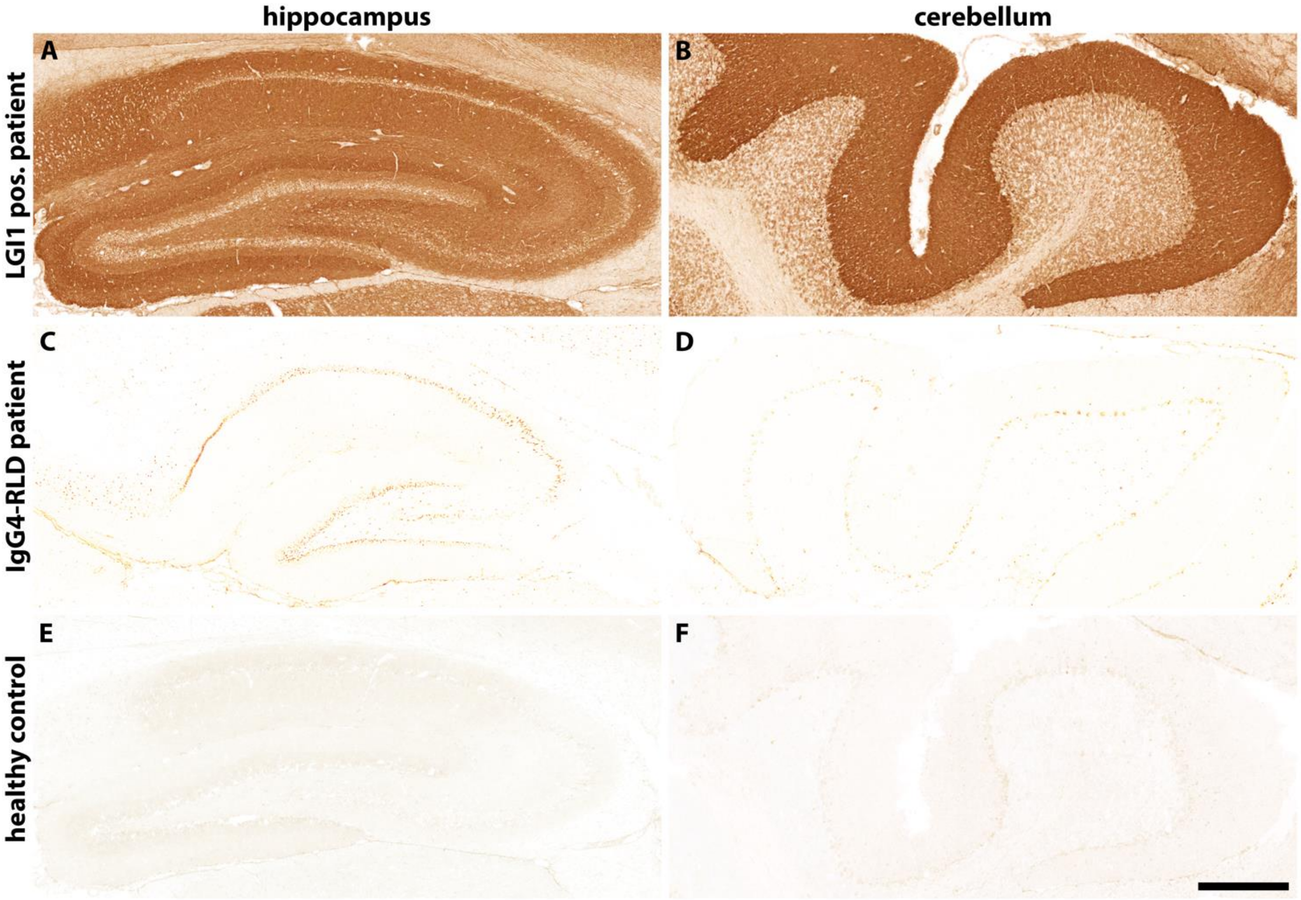

Supplementary Figure 2: Tissue-based assay for detection of anti-neuronal autoantibodies. Representative image of one IgG4-AID patient positive for LGI1 autoantibodies shows a strong neuropil staining pattern in the hippocampus **(A)** as well as in the molecular layer of the cerebellum **(B)**, whereas the neuropil of an IgG4-RLD patient **(C, D)** and a healthy control **(E, F)** remains negative. IgG4-RLD = IgG4-related disease; LGI1 = leucine-rich glioma inactivated protein- 1; pos. = positive

Supplementary Table 1: Clinical and epidemiological data of the IgG4-AID study cohort.

AChR = acetylcholine receptor; AIE = autoimmune encephalitis; approx. = approximately; Aza = azathioprine; Caspr1 = contactin-associated protein-like 1; Caspr2 = contactin-associated protein-like 2; CIDP = chronic inflammatory demyelinating polyneuropathy; CNTN1 = contactin 1; CSF = cerebrospinal fluid; d = day; F = female; GBS = Guillain-Barré syndrome; HDMP = high dose methylprednisolone; IVIg = intravenous immunoglobulins; i.v. = intravenous; LE = lower extremities; LGI1 = leucine-rich glioma inactivated protein- 1; M = male; MG = myasthenia gravis; min = minutes; MRI = magnetic resonance imaging; mRS = modified rankin scale; MS = multiple sclerosis; MuSK = muscle-specific kinase; n.a. = not applicable; neg. = negative; NF155 = neurofascin155; pan-NF = positive for pan neurofascin antibodies (155/140/186); PLEX = plasma exchange; Prs = prednisolone; p.o. = per os; Pyr = pyridostigmine bromide; RTX = rituximab; sc = subcutaneous; UE = upper extremities.

patient ID	antigen	age [years]	sex	age at disease onset [years]	clinical diagnosis	clinical symptoms	autoantibody titer/score	CSF [cell/µL]	oligoclonal bands	highest mRS	acute therapy
LGI1 #1	LGI1	49	F	48	AIE	MRI-neg. focal epilepsy: several episodes of blurry vision, general weakness, impaired mentation of approx. 10-15 min duration per day, approx. 7 episodes of electrifying paresthesias and generalized goose bumps of 3-4 seconds duration per day	1:6400	n.a.	n.a.	1	lamotrigine 100 mg/d
LGI1 #2	LGI1	69	M	69	AIE	anxiety, cognitive impairment, autonomic disturbance, stereotypical behavior	-	3	neg.	2	HDMP, IVIg, RTX, Prs p.o.
LGI1 #3	LGI1	49	M	49	AIE	fasciculations of the lower leg, dysesthesia of the lateral thigh area, insomnia, memory impairment	-	2	n.a.	1	HDMP, IVIg
LGI1 #4	LGI1	63	F	63	AIE	anxiety, seizures, cognitive impairment, nausea	1:6400	2	neg.	1	IVIg, RTX
LGI1 #5	LGI1	54	M	54	AIE	psychosis, anxiety, insomnia, social withdrawal, seizures, cognitive impairment, dyskinesia, autonomic disturbance, recurrent diarrhea for few weeks to months	1:6400	2	neg.	2	HDMP, IVIg, RTX, Prs p.o.
LGI1 #6	LGI1	45	M	45	AIE	psychosis	1:3200	7	neg.	1	HDMP, RTX, Prs p.o.
LGI1 #7	LGI1	55	M	55	AIE	reduced vigilance, cognitive impairment, autonomic disturbance	1:3200	0	n.a.	1	IVIg, Prs p.o.
LGI1 #8	LGI1	62	M	62	AIE	psychosis, anxiety, insomnia, social withdrawal, cognitive impairment	1:3200	3	n.a.	2	HDMP, Prs p.o.
LGI1 #9	LGI1	68	M	68	AIE	cognitive impairment	1:3200	1	neg.	1	Prs p.o.
LGI1 #10	LGI1	62	F	62	AIE	MRI-neg. focal epilepsy, cognitive impairment, depression, hallucinations, frequent subclinical temporal lobe seizures, pressing occipital headaches of location, lightheadedness, general weakness, impaired mentation, frequent trance-like episodes with formed visual hallucinations, jerking of right UE, staring gaze, oral automatisms and altered breathing pattern of few seconds duration	1:3200	0	neg.	3	HDMP, 2 cycles of IVIg, 4 cycles of RTX
LGI1 #11	LGI1	59	M	59	AIE	social withdrawal, autonomic disturbance, cramps, fasciculations, myokymia	1:1600	13	neg.	1	IVIg, Prs p.o.
LGI1 #12	LGI1	73	F	72	AIE	memory disturbances, confusion, epilepsy, inattention, hyponatremia of 118 mmol/L	1:6400	0.3	n.a.	n.a	valproate, quetiapine, HDMP, IVIg
LGI1 #13	LGI1	68	M	68	AIE	focal seizures, insomnia, memory impairment, pallhypesthesia, hyporeflexia, cognitive impairment, personality change	1:1600	26	neg.	0	HDMP, RTX, Prs p.o.
LGI1 #14	LGI1	65	M	65	AIE	agitation, insomnia, memory and language disturbances, confusion, epilepsy, hyponatremia of 116 mmol/L	1:3200	1	n.a.	n.a.	escitalopram 10 mg/d, sodium replacement, levetiracetam 1000 mg/d, lacosamide 200 mg/d, HDMP
LGI1 #15	LGI1	59	M	59	AIE	focal seizures, vertigo symptoms, epigastic auras	1:6400	1	neg.	0	HDMP, IVIg
Caspr2 #1	Caspr2	65	M	65	AIE	seizures, speech disturbance	1:1600	3	neg.	1	IVIg, RTX, Prs p.o.
Caspr2 #2	Caspr2	67	M	66	small fiber neuropathy	neuropathic pain	1:400	n.a.	n.a.	n.a	-
Caspr2 #3	Caspr2	78	M	78	AIE	limbic encephalitis	1:1600	n.a.	n.a.	n.a.	-
Caspr2 #4	Caspr2	69	M	69	AIE	reduced vigilance, cognitive impairment, dyskinesia	1:6400	12	n.a.	3	HDMP, Prs p.o.
Caspr2 #5	Caspr2	53	M	53	AIE	psychosis, memory deficits, sensory ataxia with neuropathic pain	1:12800	4	neg.	2	HDMP, Prs p.o.
Caspr2 #6	Caspr2	68	M	68	AIE	cognitive impairment, organic psychosyndrome	1:12800	0	neg.	0	HDMP, RTX, PLEX, Prs p.o.
Caspr2 #7	Caspr2 + LGI1 + AChR	69	M	68	AIE	behavioral, psychiatric and cognitive disturbances, movement disorder, tremor, severe episodes of pain on the extremities	1:3200	1	neg.	n.a.	thymectomy
Caspr2 #8	Caspr2	20	M	19	AIE	agitation, confusion, dyskinesia, abnormal posture, orofacial dyskinesia, autonomic instability, sleep impairment, heteroaggressivity, auditory hallucinations, mood alteration, carrier of membranous nephropathy since 2016	1:1600	0.3	n.a.	n.a.	no immunosuppressive therapy
Caspr2 #9	Caspr2	32	F	30	AIE	rare myokymias, fasciculations, cramps	1:3200	n.a.	n.a.	n.a.	thymoma (8 years ago) treated with surgery, chemotherapy and radiotherapy / no immunosuppressive therapy
NF155 #1	NF155	24	M	24	CIDP with minimal symptoms	sensory motor neuropathy of UE and LE, tremor	-	10	yes	1	IVIg
NF155 #2	NF155	10	M	9	CIDP	ataxia, tremor, sensory deficits of UE and LE	-	21	neg.	5	IVIg, RTX (two 1g doses 2 weeks apart), PLEX, Prs p.o.
NF155 #3	pan-NF155/140/186	56	M	56	GBS + MS (since 2010) + Graves´ disease	tetraparesis, sensory motor deficits of UE and LE; death	1:40960	1	n.a.	5	IVIg, RTX (two 1g doses 2 weeks apart), PLEX
CNTN1 #1	CNTN1 + Caspr1	75	M	74	CIDP	oculomotor nerve paresis, ptosis, ataxia, diplopia, dysphagia, tetraparesis, intubation; death	1:3200	n.a.	n.a.	n.a.	IVIg, PLEX
CNTN1 #2	CNTN1 + Caspr1	54	M	54	CIDP	senso-motoric neurological deficits of UE and LE, sensory ataxia, dysesthesia, edema (ascites and leg edema), hypoproteinemia, hypoalbuminemia, proteinuria	-	normal (<5)	neg.	n.a.	i.v. methylprednisolone (4x 250 mg and 3x 125 mg, over 7 d), followed by Prs p.o.
CNTN1 #3	CNTN1	72	M	72	CIDP	-	1:200	n.a.	n.a.	n.a.	3 cycles of IVIg
CNTN1 #4	CNTN1	59	M	59	CIDP	-	1:200	n.a.	n.a.	n.a.	IVIg
CNTN1 #5	CNTN1	72	M	72	acute onset CIDP	-	1:1600	n.a.	n.a.	n.a.	-
CNTN1 #6	CNTN1	82	M	82	CIDP	gait disorder, paresthesias in stocking-glove distribution, intention tremor, weak tendon reflexes UE, absent tendon reflexes LE	1:160	5	neg.	4	2 cycles of IVIg
CNTN1 #7	CNTN1	72	M	68	CIDP	ascending paraparesis, diplopia, facial paralysis right	1:1280	4	n.a.	0	IVIg, RTX, PLEX
CNTN1 #8	Caspr1	82	M	83	CIDP	tetraparesis, stocking-glove distribution hypesthesia, absent tendon reflexes	1:1600	6	neg.	5	3 cycles of IVIg, PLEX (1 cycle of 5 sessions), RTX
MuSK #1	MuSK	62	F	41	generalized MG (initially only ocular symptoms)	-	4	-	-	0	thymectomy / Pyr, Prs, Aza
MuSK #2	MuSK	32	F	24	generalized MG (initially only ocular symptoms)	ophtalmoparesis, generalized muscle weakness	3.5	-	-	1	Pyr, Prs, Aza, RTX
MuSK #3	MuSK	42	F	38	generalized MG	dysarthria, dysphagia, ophtalmoparesis, ptosis, facial muscle weakness	2.5	-	-	2	RTX
MuSK #4	MuSK	44	M	44	generalized MG	-	2	-	-	0	Pyr, Prs, Aza
MuSK #5	MuSK, borderline	36	M	34	ocular MG	-	2	-	-	1	Prs p.o.
MuSK #6	MuSK	58	M	58	generalized MG	ptosis, ophtalmoparesis, proximal muscle weakness, dysarthria, dysphagia	2	-	-	n.a.	Pyr, Prs, IVIg, RTX, Ibrutinib
MuSK #7	MuSK	27	F	27	generalized MG	orofacial muscle weakness, dysarthria	4	-	-	1	-
MuSK #8	MuSK	40	F	32	generalized MG	dysarthria, dysphagia, ophtalmoparesis, ptosis, orofacial and extremity muscle weakness, dyspnea	4	-	-	2	thymectomy / Pyr, Prs, sc IG, RTX
MuSK #9	MuSK	35	F	35	generalized MG	ophtalmoparesis, dysphagia, proximal muscle weakness	3.5	-	-	1	Pyr, RTX
MuSK #10	MuSK	28	F	28	generalized MG	dyspnea, dysphagia, dysarthria, proximal muscle weakness	1.5	-	-	2	thymectomy / Pyr, Prs, Aza, RTX
MuSK #11	MuSK	50	F	25	generalized MG	-	4	-	-	2	thymectomy / Prs, IVIg
MuSK #12	MuSK	37	F	47	generalized MG	dysarthria, dysphagia, ophtalmoparesis	4	-	-	2	Prs, RTX
MuSK #13	MuSK	57	F	21	generalized MG	-	0.5	-	-	2	thymectomy / Prs p.o.
MuSK #14	MuSK	42	F	36	ocular MG	-	0.5	-	-	1	Pyr, Prs p.o.
MuSK #15	MuSK	11	F	3	generalized MG	-	0.5	-	-	1	thymectomy/ IVIg, Prs p.o., cyclosporine

Supplementary Table 2: Epidemiological, clinical, serological, and histopathological data of the IgG4-RLD study cohort. None of the IgG4-RLD patients had an immunotherapy before blood sampling. An immunosuppressive agent was administered in 4/19 IgG4-RLD patients before blood sample taking. Increased IgG4 plasma cell infiltration indicates either the IgG4:IgG ratio and/or $IgG4^{+}$ cells per high power field. F = female; IgG4 = immunoglobulin type G subclass 4; IgG4-RLD = IgG4-related disease; M = male; Prs = prednisolone; p.o. = per os.

patient ID	age [years]	sex	age at disease onset [years]	time from disease onset until blood sampling [months]	date of diagnosis [month/year]	time difference between blood sampling and date of diagnosis [months])	acute therapy including immunosuppression and immunotherapy (before blood sampling)	clinical phenotype	serological features (IgG4 concentration [g/L]) reference value (normal: <1.35 g/L)	histopathological features (increased IgG4 plasma cell infiltration (1), storiform fibrosis (2), obliterative phlebitis (3))
IgG4-RLD #1	76	F	76	2	06/2019	-6	Prs p.o.	IgG4-related pachymeningitis	0.87	(1), (2)
IgG4-RLD #2	48	F	48	7	05/2015	-1	no	IgG4-related pseudotumor orbitae	0.90	(1), (2)
IgG4-RLD #3	70	M	70	3	07/2017	0	no	IgG4-related chronic sclerosing sialadenitis (parotid gland)	17.10	(1)
IgG4-RLD #4	62	M	62	1	12/2019	-1	no	IgG4-related pancreatitis and sialadenitis (parotid gland)	7.03	(1), (2)
IgG4-RLD #5	48	F	47	3	08/2016	0	no	IgG4-related pseudotumor orbitae	0.79	(1), (2)
IgG4-RLD #6	64	F	63	2	07/2020	0	no	IgG4-related chronic sclerosing sialadenitis (submandibular gland)	0.99	(1), (2)
IgG4-RLD #7	59	F	48	124	03/2015	-4	no	IgG4-related chronic dacryoadenitis, pancreatitis and sialadenitis	12.10	(1)
IgG4-RLD #8	76	M	75	7	12/2016	2	no	IgG4-related chronic sclerosing sialadenitis (submandibular gland)	5.22	(1), (2)
IgG4-RLD #9	69	F	68	8	04/2018	-1	no	IgG4-related dacryoadenitis	6.23	no
IgG4-RLD #10	71	M	68	42	12/2015	40	no	IgG4-related pseudotumor orbitae	2.11	(1), (2)
IgG4-RLD #11	50	F	50	4	06/2017	-2	no	IgG4-related pseudotumor orbitae	0.44	(1), (2)
IgG4-RLD #12	57	M	57	0	04/2019	0	no	IgG4-related sialadenitis (parotid gland)	1.04	(1), (2)
IgG4-RLD #13	36	F	31	64	06/2019	-1	no	IgG4-related sclerosing thyroiditis	0.44	(1), (2)
IgG4-RLD #14	68	M	62	71	01/2016	0	Prs p.o.	IgG4-related cholangitis, pancreatitis and sialadenitis (parotid gland)	16.70	(1)
IgG4-RLD #15	28	F	23	66	06/2020	0	no	IgG4-related chronic sclerosing laryngitis	0.98	(1), (2), (3)
IgG4-RLD #16	14	M	12	27	04/2019	-12	Prs p.o.	IgG4-related sialadenitis (parotid gland)	4.17	no
IgG4-RLD #17	46	M	47	-7	10/2020	-12	no	IgG4-related aortitis, pseudotumor orbitae and sialadenitis	2.46	(1), (2)
IgG4-RLD #18	36	F	36	8	12/2017	-12	Prs p.o.	IgG4-related pseudotumor orbitae	0.56	no
IgG4-RLD #19	90	M	90	0	03/2019	0	no	IgG4-related dacryoadenitis and pseudotumor orbitae	12.10	no
